# “Cookbook medicine”: Exploring the impact of opioid prescribing limits legislation on clinical practice and patient experiences

**DOI:** 10.1016/j.ssmqr.2023.100273

**Published:** 2023-06

**Authors:** Elizabeth Joniak-Grant, Natalie A. Blackburn, Nabarun Dasgupta, Maryalice Nocera, Samantha Wooten Dorris, Paul R. Chelminski, Timothy S. Carey, Shabbar I. Ranapurwala

**Affiliations:** a University of North Carolina Injury Prevention Research Center, 725 Martin Luther King Blvd., Chapel Hill, NC, 27599-7505, USA; b Department of Health Behavior, Gillings School of Global Public Health, University of North Carolina at Chapel Hill, 135 Dauer Drive, Chapel Hill, NC, 27599-7440, USA; c Office of Research, Innovations, and Global Solutions, Gillings School of Global Public Health, University of North Carolina at Chapel Hill, 135 Dauer Drive, Chapel Hill, NC, 27599-7415, USA; d Departments of Allied Health Sciences and Medicine, School of Medicine, University of North Carolina at Chapel Hill, Chapel Hill, NC, 27599, USA; e Department of Medicine, School of Medicine, Cecil G. Sheps Health Center for Services Research, University of North Carolina at Chapel Hill, Chapel Hill, NC, 27599, USA; f Department of Epidemiology, Gillings School of Global Public Health, University of North Carolina at Chapel Hill, 135 Dauer Drive, Chapel Hill, NC, 27599-7435, USA

**Keywords:** Drugs/medication, Medical practice, Pain, Regulation, Qualitative/in-depth interviews, North America

## Abstract

Opioid dependence and overdose are serious public health concerns. States have responded by enacting legislation regulating opioid-prescribing practices. Through in-depth interviews with clinicians, state officials, and organizational stakeholders, this paper examines opioid prescribing limits legislation (PLL) in North Carolina and how it impacts clinical practice. Since the advent of PLL, clinicians report being more mindful when prescribing opioids and as expected, writing for shorter durations for both acute and postoperative pain. But clinicians also report prescribing opioids less frequently for acute pain, refusing to write second opioid prescriptions, foisting responsibility for patient pain care onto other clinicians, and no longer writing opioid prescriptions for chronic pain patients. They directly credit PLL for these changes, including institutional policies enacted in response to PLL, and, to a lesser degree, notions of “do no harm.” However, we argue that misapplication of and ambiguities in PLL along with defensive medicine practices whereby clinicians and their institutions center their legal interests over patient care, amplify these restrictive changes in clinical practice. Clinicians’ narratives reveal downstream consequences for patients including undertreated pain, being viewed as drug-seeking when questioning opioid-prescribing decisions, and having to overuse the medical system to achieve pain relief.

## 1. Introduction

Opioid dependence and overdose are serious public health concerns in the United States. Opioid overdoses rose significantly with the tripling of prescription opioids from the 1990s–2000s (Paulozzi et al., 2006). 2013 marked the first decrease in opioid prescription rates and this trend continued in North Carolina and nationally (Centers for Disease Control, 2021). Yet opioid overdose deaths continue to rise as heroin, polysubstance use, and fentanyl later supplanted prescription opioids as chief causal agents (Ciccarone, 2019; NCDHHS, 2017). In response and built on the assumption that opioid misuse and overdose are consequences of continued overprescribing (Netherland & Hansen, 2016), nearly ¾ of US state legislatures have passed opioid prescribing limits legislation (PLL) (McGinty et al., 2022). These legislative responses are fueled by media coverage and policy shifts favoring punitive solutions such as government intervention, policing, and professional regulation of physicians (Webster et al., 2020) and call for increased surveillance of clinicians and their prescribing practices, limiting clinician autonomy (Knight et al., 2017; Netherland & Hansen, 2016).

North Carolina’s (NC) PLL, which is part of the Strengthen Opioid Misuse Prevention (STOP) Act (2017), took effect January 1, 2018 and places a five and seven-day limit on initial opioid prescriptions for acute and postoperative pain, respectively, for opioid-naïve patients. Clinicians are permitted to write a second prescription with no limitations. Other elements of the STOP Act include mandates that call for clinicians to electronically prescribe opioids and use the state’s prescription drug monitoring program.

Although these laws are widespread, little attention has been paid to contextual factors related to opioid-prescribing by clinicians (Knight et al., 2017) and how these laws impact practitioner attitudes and clinical practices (Stone et al., 2020). Early findings suggest laws and recommendations can induce a fear of prescribing opioids (Lynch & Katz, 2017) and create confusion about to which patients the law applies (Chua et al., 2020).

Thus, we conducted in-depth interviews with clinicians and other stakeholders to explore clinician and institutional responses to and implementation of PLL in NC. We also examine clinicians’ perspectives and experiences related to downstream consequences on patients and patient care.

## 2. Methods

### 2.1. Recruitment and sample selection

We conducted in-depth, semi-structured interviews with 54 participants between June 2019 and February 2020, including 16 official and organizational stakeholders and 38 clinicians. This study was approved by the Institutional Review Board at University of North Carolina, Chapel Hill (IRB# 18–2437) as part of a larger evaluation of the STOP Act (Maierhofer et al., 2021).

Officials and organizational stakeholders were involved in the development and/or passage of PLL. They were recruited through purposive sampling based on the team’s knowledge of the legislation and through snowball sampling in hopes of interviewing representatives from all principal organizations. Respondents represented government (n = 4), professional associations (n = 5), advocacy groups (n = 3), regulators (n = 2), and substance use treatment organizations (n = 2).

Clinicians were recruited three ways. First, we randomly sampled emergency medicine, obstetrics and gynecology, orthopedic, and otolaryngology clinicians from the NC Medical Board (NCMB) licensing database. These specialties were selected for having the highest rates of opioid-prescribing in NC (Ringwalt et al., 2014). We also worked with statewide professional medical associations to share our study details and contact information on their listservs and in newsletters. Third, we utilized snowball sampling, inviting participants to share study details with friends and colleagues. Approximately 15% of clinicians were recruited through the NCMB licensing database, 65% through statewide professional associations, and 20% through snowball sampling.

Clinicians, defined as medical doctors (n = 21), nurse practitioners (n = 2), or physician assistants (n = 15), were located throughout NC and worked in a range of specialties including primary care, internal medicine, obstetrics and gynecology, orthopedics, and emergency medicine from 1 to 40 years. Inclusion criteria required an active NC medical license and prescribing opioid analgesics for acute and/or postoperative pain at least once in the preceding 5 years.

### 2.2. Data collection

Our two interview guides, one for officials and stakeholders and the other for clinicians, were informed by study objectives, Consolidated Framework for Implementation Research (CFIR) domains such as “characteristics of individuals” (e.g., knowledge and beliefs about the intervention) and “intervention characteristics” (e.g., complexity) (Damschroder et al., 2009), literature reviews, previous work by team members, and one team member’s lived experience as a chronic, non-cancer pain patient. Topics of inquiry for officials and stakeholders included the development and goals of PLL, personal and organizational motivations, and the major players; topics for clinicians included PLL implementation, communications with colleagues and patients, resources, their beliefs regarding the strengths and weaknesses of PLL, enforcement of PLL, and opioid prescribing more generally.

Interview guides were used to orient the interviews, while our semi-structured approach allowed tailored follow-up questions. Questions were added, removed, and refined throughout the interview process to reflect our on-going analysis and to pursue new lines of inquiry, in line with grounded theory (Charmaz, 2014).

NAB, a white, cisgender woman, with qualitative interview training and a terminal public health degree conducted interviews. Roughly half were conducted in-person and the remaining by phone. Informed consent was obtained, and all interviews were audio-recorded and professionally transcribed. Interviews lasted 45 min to 1 h and participants were offered a $50 cash gift card, which some declined. Data collection with state officials and stakeholders ceased once we interviewed all key individuals who would participate. Data collection with clinicians ceased when we reached saturation for themes related to the study’s objectives and with the advent of the COVID-19 pandemic in the United States.

### 2.3. Data analysis

Data coding and analysis utilized flexible coding (Deterding & Waters, 2018) informed by a modified grounded theory approach (Charmaz, 2014). EJG and NAB closely read several initial interviews and analyzed data early on through open, line-by-line coding, memoing independently, and then discussing observations to assess interview topics and note emerging and recurring patterns.

Once data collection was complete, EJG developed preliminary codebooks that consisted of inductive index codes (Deterding & Waters, 2018) based on early open coding and deductive index codes based on interview guides and study objectives. EJG and NAB then used Dedoose (v9.0.17, Los Angeles, California) to co-code 5% of interviews, reconciling any coding differences through discussion. Once inter-coder agreement was strong, they individually index-coded remaining interviews. Excerpts with coding ambiguity were jointly reviewed and coded.

EJG then isolated all excerpts linked to the index codes most pertinent to the focus of this paper and conducted line-by-line open analytic coding. Memoing was used to explore emerging concepts and recurring themes, noting areas of similarity and difference (Emerson et al., 2011). Memos and exemplary data excerpts were shared with team members to decide which themes to pursue and consider possible explanations. The team then returned to the literature to further inform our analysis and begin to place our concepts and themes within it (Strauss & Corbin, 1998). Negative cases—those that *countered* our explanations and conceptual framework—were given particular attention and incorporated for more nuanced understanding.

As themes emerged, it became apparent that clinicians’ opioid-prescribing practices are impacted by individual, interpersonal, cultural, and larger social forces. Thus, to help us better contextualize opioid-prescribing practices, our analysis and interpretations were informed by the social ecological model (SEM), social medicine, and writings on stigma.

The SEM maintains that health behavior and outcomes are impacted by the interplay of factors at five levels of influence, namely intrapersonal, interpersonal, institutional, community, and public policy (Golden & Earp, 2012). Like the SEM, social medicine moves beyond disease conceptualizations that locate health problems and solutions within individuals to focus on the social origins of illness (Waitzkin et al., 2001). Social medicine works to understand health and health care by examining clinicians’ beliefs, current science, and the impact of social and political forces on the relationship between clinicians and patients, patients’ beliefs and experiences, the culture of medicine, and social determinants (e.g., economic, political, legal, cultural) of disease (Stonington & Holmes, 2006). Thus, to understand opioid-prescribing under NC’s prescribing limits legislation (PLL), we explored the development of PLL, clinicians’ beliefs, attitudes, and perceptions about opioids, PLL, and clinician-patient interactions, clinician and institutional responses to PLL, and the culture of medicine as it relates to opioid-prescribing and legal liability.

This exploration would be incomplete, however, without considering how opioid-related stigma impacts PLL and opioid-prescribing practices. Stigma is an attribute or difference that is deeply discrediting or is labelled as such by those with power, is typically associated with a series of negative characteristics, and often leads to stereotyping, status loss, and discrimination at interpersonal and structural levels (Goffman, 1963; Link & Phelan, 2001). Opioids, patients who use opioids, and clinicians who prescribe them are stigmatized (Knight et al., 2017; McCradden et al., 2019). This stigmatization is fueled by pain’s subjective nature and clinicians’ tendency to question its’ legitimacy (Webster et al., 2019), the link between opioids and fears of addiction, and clinicians who view patients as potentially playing a game to access opioids (Crowley-Matoka & True, 2012).

### 2.4. Use of quotes

Indented stand-alone paragraphs or quotation marks embedded in text indicate direct quotes from respondents. All quotes were chosen as exemplars of respondents’ attitudes, experiences, and/or practices unless noted otherwise.

## 3. Results

To provide context, this section begins by describing the development of prescribing limits legislation (PLL) through interviews with officials and organizational stakeholders. Then, through interviews with clinicians, we explore the impacts of PLL on opioid-prescribing practices, clinical practice, and ancillary impacts on chronic non-cancer pain management. We highlight previously undescribed institutional responses to PLL that further restrict opioid-prescribing, and examine how misconceptions about, reframing of, and ambiguities in PLL influence clinical practice. Finally, we explore clinician perspectives regarding downstream consequences on patients and highlight the role defensive medicine plays in these outcomes.

### 3.1. Background: developing prescribing limits legislation

PLL was rooted in beliefs by the NC legislature and other organizational stakeholders that the opioid epidemic is caused and sustained by patients developing opioid use disorder after seeking legitimate pain treatment:

We hear over and over again of people who had an injury, an acute pain episode, and then they started using opioids that way and then got hooked, and then moved to heroin. So it’s not like a scientific study of that’s how people got into their addiction, but it’s very obvious. (Government Official).

References to “addiction” and the high risk of getting “hooked” to doctor-prescribed opioids were abundant in interviews and help explain why those who developed PLL saw it as a path to decrease opioid-induced deaths, misuse, dependency, and diversion (e.g., sharing or selling prescription medications). They viewed PLL as a starting point to stop what they believed was a clinical habit of overprescribing:

So a lot of doctors were just in the habit of writing scripts for like 30 days, 30 days, 30 days … we felt like we need to break that habit. (Government Official).

Initial legislative drafts and discussions also included day limits for chronic pain. However, after resistance from professional association representatives, “chronic pain was off the table” (Advocacy Stakeholder) although advocacy representatives maintained “PLL was going to affect pain patients.”

Legislative authors of PLL believed the 5- and 7-day limits allowed patients enough days to “get the pain relief they needed” (Government Official) without requiring a prescription over the weekend when getting a prescription written and filled is more difficult:

So the number of days ended up being that thing that we could all end up agreeing on … because it had the simplicity attached to it and had enough days for acute pain that you didn’t get somebody hung out over a weekend. (Professional Association Member).

Further, clinicians may write a second prescription without limits if a patient would benefit from continued opioid analgesia (commonly referred to as a “refill”). These second prescriptions played an important part in PLL’s conceptualization:

[In developing prescribing limits my thoughts were on] getting it right. Not being too limiting, having the … ability to have the prescriber prescribe additional opioid pain relief if they [patients] needed it. If it was justified. (Professional Association Representative).

Yet, while some stakeholders sought balance to ensure opioids were still available, others hoped PLL would lead to even shorter durations and fewer opioid prescriptions because they believed any opioid prescription could lead to opioid dependence:

The more days you’re on opioids initially … the more likely you will wind up with addiction. (Government Official).

This need for explicit justification, the undercurrent of addiction fears, and some stakeholders’ desire for severe restrictions, complicated clinician and institutional responses to PLL.

### 3.2. Impacts on clinical practice

#### 3.2.1. Clinicians prescribe opioids to fewer acute pain patients and for shorter durations

Clinicians report that PLL pushes them to be more mindful about when, why, and how much they prescribe, as well as the associated risks. They report prescribing opioids to fewer acute pain patients with some abstaining altogether:

Honestly, we’ve changed so much in our practice that very, very few people that we’re seeing in the outpatient setting get any opioids. So even people now that come in that have broken wrists, broken collar bones, broken ankles, they’re getting Tylenol and ibuprofen. (Family Physician MD).

When providers prescribe opioids, they often prefer prescribing to postoperative patients or those with visual and measurable signs of pain (e.g., broken bone) versus to those with only symptoms (e.g., severe pain):

I very, very, very rarely ever give them [opioids] for any kind of like GI complaints, or like abdominal pains and things like that … the ones I typically would give opioids for postop are typically orthopedic. (Emergency Medicine PA).

In addition, clinicians detail writing opioid prescriptions for shorter durations for both acute and postoperative patients:

Prior to the STOP Act, I wasn’t as hard and fast about the duration so [I’d prescribe] maybe a couple weeks. Now I’m much more mindful of sticking to five to seven days because of our legislation. (Internal Medicine PA).

While this is not surprising, clinicians also note regularly prescribing for acute pain for two to three days (e.g., “I find that three days is kind of long enough to get them to a specialist or reevaluate,” Family Medicine MD). Writing opioid prescriptions for a maximum of three days and then referring out or advising follow-up with another care provider is a recurring pattern.

#### 3.2.2. Second prescriptions: requiring consultations, refusal to write, and passing the buck

Before PLL, clinicians could write multiple prescriptions for Schedule II narcotics (e.g., oxycodone) during the initial consultation up to a 90-day supply (Gabay, 2013). PLL now requires clinicians to conduct “a subsequent consultation for the same pain” (STOP Act, 2017, pp. 4). What constitutes a consultation is undefined. Clinicians believe a chart review or phone call with the patient is likely sufficient under the law, yet nearly all who wrote second prescriptions require in-person appointments.

Clinicians who report never writing second prescriptions frequently pass the buck, avoiding action by foisting responsibility for patient pain management onto another clinician (Ashforth & Lee, 1990). Surgeons routinely redirect patients seeking second prescriptions to emergency departments and primary care providers, frustrating clinicians:

[Post-op patients are] calling me on Friday; they had surgery on Monday, [are] anticipating pain over the weekend, and their surgeon says, “I’ve given you the five days. I don’t think you need more pain [meds].Call your PCP.” That’s the most frustrating … it just gets dumped to us. (Internal Medicine MD).

These referred-to providers feel ill-equipped to decide whether a second prescription is warranted, what would be an appropriate dose and amount, and if ongoing pain signals surgical complications. As a result, referred-to primary care and family medicine providers express a general reluctance to write second prescriptions:

I would not say it [writing a second prescription for post-op patients] is never, but it’s just very infrequent because a majority of the time I feel like if somebody is having postoperative pain to the level where they feel like they need more opioids, I feel like they need to be talking to that surgeon because that to me is a red flag that the postoperative healing process could not be going as planned. (Family Physician MD).

Just as surgeons routinely pass the buck for second opioid prescriptions to other clinicians, primary care physicians, nurse practitioners, and physician assistants note they have responded to PLL by increasingly referring patients to specialists (e.g., orthopedics, pain medicine):

I guess because of the prescribing limits I’m more likely to say, “Go to the right person right away” versus, “Come to me, get pain medicine and then see how you feel in a couple of days to a week and then go see ortho.” I might just say, “Go see ortho.” (Internal Medicine MD).

This excerpt demonstrates how PLL has caused some clinicians to view previous conservative measures (i.e., waiting a week and taking opioids) as now risky. Risk perceptions play an important part in PLL implementation.

#### 3.2.3. Clinicians do not prescribe opioids to and refuse to treat chronic non-cancer pain patients

When asked how PLL impacts their opioid-prescribing patterns with *acute* and *postoperative* pain patients, clinicians consistently volunteer that they no longer prescribe for chronic pain, rarely distinguishing between chronic non-cancer pain (CNCP) and cancer pain although their comments insinuate presumptions that cancer-related pain is treated by oncology teams:

We have our own opioid initiative as well. Specifically, we are limited to prescribing patients 10 pills … and we don’t prescribe chronic pain medication. (Emergency Medicine MD).

We’re one of the few places that will actually take people on opiates now. A lot of private practice docs are just not wanting to see those folks. (Family Medicine MD).

Instead of treating CNCP patients, clinicians note they routinely refer patients to pain management.

Some clinicians credit PLL for their refusal to prescribe opioids for chronic pain:

It’s all because of the STOP Act and since the whole crackdown started, we tell patients we do not provide long-term opioid medication. (Orthopedic PA).

Others see these refusals as the end-result of an ongoing downward trajectory in opioid-prescribing accelerated by the STOP Act.

### 3.3. The role of PLL in changing clinical practice

While some clinicians describe PLL as the “key driver” of their opioid-prescribing practices, others view it as a contributing factor. Beyond PLLs, clinicians mention national guidelines and directives, institutional and departmental policies, cultural shifts among colleagues, patient willingness to try non-opioid modalities, personal concerns about opioid misuse, overdose, and diversion, and their desire to “do no harm.”

Clinicians’ statements regarding their opioid-prescribing practices are consistently peppered with comments that cast opioids as inherently high-risk. They tell stories of “good” patients who quickly became “addicted” to prescribed opioids:

Before I would have been tempted to give them extra, give them plenty, so that they wouldn’t run out. Now I want them to run out and contact me again. And I want them to transition to something else, such as Tylenol or anti-inflammatories quicker. Some of that is the STOP Act. Some of it is … A young dad who was given Tylox after a wrist fracture, he said, really, by the third day, after that, he felt emotionally addicted to it. He’s really struggled. Some of it has been a big impact from that patient. (Internal Medicine MD).

This excerpt demonstrates clinicians’ recognition of PLL as one factor impacting their opioid-prescribing. It also highlights the impact of patient narratives; in this case, a “young dad” who became “emotionally addicted” in three days.

### 3.4. Institutional responses to PLL further restrict opioid prescriptions

In interviews, clinicians discuss how institutions responded to PLL by establishing more stringent limits. Clinicians note that their organizations and departments often mandated even shorter day limits soon after PLL went into effect, prohibited opioid prescriptions for acute and/or chronic pain (e.g., “the higher-ups don’t really want chronic pain issues managed in our offices”), prohibited second prescriptions, or permitted second prescriptions only under certain conditions (e.g., for one’s own patients during office hours). A clinician discussed how their organization’s response to PLL has changed “everything about” how they treat CNCP patients despite PLL only limiting acute and postoperative opioid prescriptions:

[Before PLL] we could see patients when we felt it was appropriate. Now … particular information has come from [our larger organization] about how they want prescribers to manage chronic pain patients. For example, how many times you need to see them. Contracts, writing up contracts that we’re supposed to use. Frequency of urine drug testing. How many prescriptions you can write, etcetera. (Internal Medicine MD).

This excerpt demonstrates clinicians’ awareness that these policies began shortly after PLL was enacted and highlights why they believe PLL amplified punitive responses such as contract and drug testing for CNCP patients.

Similarly, clinicians routinely mention how commercial health insurers use PLL to deny second prescriptions and limit the quantity of dispensed pills for all patients:

I’ll say dispense 42, and the insurance will only allow for dispensing 20 or something like that. (Orthopedic PA).

In fact, Blue Cross Blue Shield of NC (BCBSNC), claiming compliance with PLL, limited payment for *all* initial opioid prescriptions to seven days three months after PLL (Maierhofer et al., 2021) and later required prior authorizations for initial and subsequent immediate-release opioid prescriptions that exceeded specific quantities (BCBSNC, 2019).

Clinicians discuss the interplay of PLL, insurance requirements, and the bureaucratic burden of prior authorizations:

The amount of prior authorizations have increased [since the PLL] and a prior authorization is not just a click of a button, it’s a 45 min process … and then you’re still calling me back in five days to repeat that entire process … in two days I’m going to get a prior authorization, two days after that I’m going to get a denial. Two days after that I’m going to get an acceptance letter that I have to sign that I’m aware they had 64 morphine equivalents instead of 60. Yeah, I get it. Then in clinic I’ve got to explain to you why it took six days to get you a prescription that you were taking in the hospital anyway. Now my 15-min visit with you to go, “Yep, you’re healed.” Is now, “Yep, you’re healed, and I think I owe you an apology.” (Trauma PA).

Thus, clinicians report that writing for certain opioid prescriptions or amounts frequently comes with a heavy bureaucratic burden, including prior authorizations, appealing denials, and signing acceptance letters. This results in delayed prescriptions, patients with untreated pain, apologies, and referred-to clinicians being reluctant to engage.

Clinicians describe similar issues with pharmacies noting some pharmacists will attempt to “verify” prescriptions by contacting clinicians to confirm whether a patient is surgical or acute, questioning amounts they deem inappropriate, and/or to inquire about tapering for CNCP patients:

I think the STOP Act was certainly the catalyst for it … I mean you write a prescription and it’s like, “Well this is seven days. Were they surgical?” “Yes, they were.” You feel like saying, “It’s none of your business, fill the prescription.” It’s now a lot of people asking a lot of questions. (Surgical PA).

Again, clinicians like this surgical PA, credit PLL for the increased administrative burden of opioid-prescribing and pharmacists desire to verify prescriptions. While some prescribers appreciated these pharmacy reviews, others, like the PA above, viewed pharmacists as unnecessarily policing their opioid-prescribing.

Thus, clinicians’ narratives reveal their opioid-prescribing practices are also bound by larger institutional responses to PLL that further restrict opioid-prescribing. As a result, occasions occur when they may not treat patients as they see fit despite adhering to PLL.

### 3.5. Putting PLL into practice: misconceptions, reframing, and ambiguity in PLL

Clinicians consistently credit PLL for changes in their opioid-prescribing practices. Sometimes the influence is explicit:

Now I’m much more mindful of sticking to five to seven days because of our legislation. (Internal Medicine PA).

However, PLL also impacts clinical practice because clinicians misinterpret, reframe, or find aspects of PLL ambiguous.

Some clinicians erroneously believe PLL requires more stringent limits. These clinicians conflate statutory compliance with either default settings within the electronic health record— “the electronic health record is the alert of the STOP Act,” (Family Medicine MD) or more restrictive professional society guidelines such as the 3-day limit recommended by the NC Hospital Association and the NC American College of Emergency Physicians (2018):

The most I will prescribe is 12 tablets. And that’s the law … it’s no more than three days in the acute pain setting. (Emergency Medicine MD).

Other clinicians know the limits are 5 or 7 days but reframe them as the maximum expected amount per patient per incident rather than a starting point for pain management. They believe needing opioids for five or more days is only for patients in the most severe pain and signals a need for further clinical care:

Now they’re saying that five days is the limit and I kind of think to myself, “Most of my patients are not having the *worst* acute pain of their life, so maybe I should rethink how much pain medication they need.” (Primary Care PA).

Clinicians also mention ambiguities regarding what constitutes a second prescription and subsequent consultation requirements:

If [patients] have been treated with five days somewhere else for acute, now do I have to do five days with me, or can I prescribe longer? (Internal Medicine MD).

I’m still a little unclear [about] a re-evaluation, does the patient actually need to be seen? (Orthopedics PA).

Finally, clinicians are unsure about if and how PLL applies to chronic pain patients experiencing acute exacerbation of their pain. A handful of clinicians also incorrectly think that PLL includes day-limits for chronic pain patients with or without cancer.

### 3.6. Downstream impacts on patient care: undertreated pain, trouble, and overuse

#### 3.6.1. Undertreated pain

While providers tend to agree PLL is a “good” or “appropriate” first step, some see it as “overgeneralized” because it fails to account for the vast heterogeneity in patients’ pain responses. They note pain is complex and the “cookbook medicine” approach of PLL causes harm:

There are going to be people who will be undertreated as a result of these legislative limits. (General Surgery MD).

For example, clinicians point out some patients need to take opioids less frequently, but for longer durations. Other injuries and surgeries, such as severe burns with exposed nerve endings, are predictably more painful and last longer:

I would say 99.9% of our patients are needing that refill. [Patients] all wonder why you’re only writing for five days’ worth after they burned 75% of their body. (Burn Unit PA).

Yet PLL provides no avenue for clinicians to write longer initial prescriptions for predictable or exceptionally painful cases.

Clinicians’ responses to PLL can lead to undertreated pain when they institute more stringent opioid-prescribing limits, pass the buck, or choose to never prescribe opioids, never write second opioid prescriptions, or not treat CNCP patients. Related, clinicians discuss how institutional and organizational policies in response to PLL do not treat patients as individuals and can leave patients with inadequately controlled pain:

If I see somebody with an acute injury, like they tore an ACL and I need to get them to orthopedics, five days [of opioids] isn’t a lot because it can take a long time to get them to orthopedics. Then orthopedics is likely going to order an MRI. Then that’s going to take another week. Then, maybe they need surgery. That’s going to take some more time to get scheduled. Now, you’re looking at somebody who’s been dealing with an ACL tear for four weeks and they’ve only been allowed five days of pain medicine. (Family Practice NP).

This excerpt, for example, shows how an organizational policy banning second opioid prescriptions disregards time to diagnosis and treatment, leading to undertreated pain.

Organizational responses to PLL can also create ethical quandaries for clinicians who find their autonomy limited:

I had a 22-year-old soccer player for college, did a slide tackle or something, had a rupture, a herniated disc, and required surgery. But from the time she gets in to see the surgeon could be three, four weeks, she’s going to be in pain. Just saying, “Here you go. I can give you a three-day supply,” that’s just not right. (Physiatry PA).

Thus, this organizational policy limiting opioid prescriptions to a single three-day prescription leads to untreated pain for the patient and an ethical quandary for the clinician who must do what they believe is “just not right.”

#### 3.6.2. Troubles in clinician-patient interactions

When asked about clinician-patient interactions related to opioid-prescribing, clinicians negatively characterize patients who voice concerns about opioid prescription amounts, ask for an opioid prescription or refill, or express reluctance about seeing a specialist:

Occasionally, you’ll get a patient who gets really feisty with you and angry that you’re not just going to refill their medicine or give them as many tablets as they want. That also makes me more suspicious that this is somebody that we’re dealing with an addiction on because they’re not really accepting of the law. If you’re really in pain, then you’re really willing to go to a pain management provider who’s more equipped to handle and help you with your pain. (Family Practice NP).

As this excerpt demonstrates, clinicians describe reacting in ways that frame patient concerns and behaviors as trouble (Emerson, 2011). In interviews, clinicians consistently use stigmatizing language such as “addiction” and “drug-seeking.” They describe patients who question or complain about their opioid-prescribing practices as potentially opioid dependent and react absolutely and punitively— “you just kind of knuckle down and don’t give them anymore” (Internal Medicine MD).

#### 3.6.3. Overuse

Clinicians point out that PLL is implemented in ways that can cause patients to overuse the medical system. Overuse refers to health care services, including clinic visits and tests that are not necessary, where the potential harms are greater than the potential benefits, or evidence is lacking for benefits (Brownlee et al., 2014). PLL fosters overuse when clinical and institutional responses to it undertreat pain or rely too much on referrals forcing patients to repeatedly touch the medical system to get needed pain relief:

[I saw] this person who had this [complicated] fracture and was waiting to get in with orthopedics [to get surgery]. They can’t get in with ortho for two weeks, so they’re going to still have pretty significant pain. She was pretty much coming to us like every five days. She came in about three times. Why can’t I just prescribe her enough medication to get her to her ortho appointment? (Emergency Medicine PA).

I had a high school friend who reached out to me. It was right after the Act had been put into place. He was kind of given the seven day of post-surgical pain treatment. It ran into a weekend, and he was just asking basically, “Is there anything else I can do,” because the surgeon was like, “It’s the weekend. I can’t send anything in, so you’re going to have to go to the ER”… It was frustrating for him because it created a lot more cost and care usage because of that law. (Internal Medicine MD).

These excerpts exemplify overuse pathways. Prescribing less than the needed days and passing the buck to the emergency room for a refill caused multiple unnecessary visits. Overuse is also apparent when CNCP patients are automatically referred to pain management.

In interviews, clinicians recognize overuse harms such as financial costs to patients and undertreated pain, which can be exacerbated by wait times. To manage delays, prescribers mention instructing patients to stretch medication until they see the specialist, trying to get patients seen more quickly, or referring patients to emergency departments. Clinicians do not mention writing a second prescription without a face-to-face consultation as a solution. Thus, while potential patient harms of multiple visits are sometimes recognized, clinicians rarely report they are factors impacting their opioid-prescribing practices:

My patients, if they still need pain management after that initial five days, they have to come back and see me again. They have to pay another co-pay which might be an issue for them. (Primary Care NP).

This clinician recognizes the financial costs for their mostly cash-pay patients, yet they still require return visits for second prescriptions.

### 3.7. The role of defensive medicine in clinicians’ response to PLL

While some clinicians struggle with their inability to prescribe opioids as they see fit, others opt-in to more restrictive opioid-prescribing practices. They explain these choices by citing vague notions of patient safety and not wanting to “do harm.” However, these comments are often overshadowed by lengthy commentaries about liability and licensure concerns.

Defensive medicine centers clinicians’ and/or institutions’ legal interests (i.e., avoiding lawsuits and liability) ahead of patient care (Kapp, 2016) and includes hedging and avoidance practices. Hedging practices provide care with no benefit or the harms outweigh potential benefits (e.g., unnecessary visits, tests, prescribing unneeded medications); avoidant defensive medicine practices occur when clinicians or institutions eschew patients (e.g., CNCP patients) and practices (e.g., prescribing opioids) they perceive as risky, perhaps by referring them out, or avoiding blame through scapegoating or misrepresentation (Ashforth & Lee, 1990; Ries & Jansen, 2021).

Most clinicians who stopped or greatly limited opioid prescriptions explain PLL has caused opioid-prescribing to come with “too much liability”:

In my previous practice, the medical director just washed his hands of it [prescribing opioids]. He just refused to follow those rules [of PLL]. He just said, “Well this is just too much. It’s too much liability now.” (Family Medicine MD).

Others note the “stressful” nature of writing opioid prescriptions and their resultant “fears,” which center on questions of legality, potential law enforcement encounters, and/or licensure concerns:

I think just the law itself. You always want to make sure you’re not doing anything illegal, number one. (Primary Care NP).

I worry that if a patient comes back and wants more narcotic and I feel it’s appropriate and I give it to her, that it’s going to send some red flag and someone’s going to come knocking on my door that I’m giving too many narcotics. (Obstetric Gynecologist MD).

[There’s] the fear in every provider’s mind that, ‘I’m not going to lose my license.’ (Orthopedic PA).

These legal and licensing concerns along with ambiguities in the law help explain why some clinicians respond to PLL by instituting avoidant or hedging defensive practices. The focus becomes “dotting all the i’s and crossing the t’s” and doing what “seems appropriate” rather than what best serves the patient. Consider the practice of requiring in-person consultations for second prescriptions:

I actually don’t know if the law explicitly says that I have to see the patient to treat them with narcotics for acute pain … I just kind of said to myself, “Okay, you know what? If this is going to be regulated now, I need to … How do I put it? Just really do it in a way that seems appropriate.” (Primary Care PA).

Again, this excerpt demonstrates clinicians’ focus on legality, regulation, and compliance ahead of patient care.

Clinicians also discuss their desire to conduct exhaustive searches in case they missed something:

I think when I heard that the prescribing limits were five days for acute pain, I kind of challenged myself to say, “I want to do better than that …” And so, I just kind of set a new standard for myself like, “Okay, after three days I want to know what’s going on.” (Primary Care PA).

This PA emphasizes “challenging” themselves, wanting to “do better” and to know “what’s going on.” Patient needs are secondary, echoing others’ comments. Thus, while some clinicians require return visits for patient care, others are focused on what they, as clinicians, want or feel looks most appropriate.

Defensive practices are also apparent in clinicians’ handling of patient trouble related to opioid-prescribing. Clinicians routinely bolster their stance by referencing and sometimes misrepresenting PLL. Misrepre-sentation is a defensive behavior wherein one avoids blame by consciously or unconsciously distorting, embellishing, or withholding information (Ashforth & Lee, 1990). Clinicians spoke at length about using PLL as a “defense,” “buffer and backbone,” “mediator,” or “muscle” to “hide behind” and manage actual or expected patient “push back” on opioid-prescribing decisions:

I might [also] lie to them and I tell them I can only prescribe in five-day increments (Trauma PA).

Thus, this clinician, and others misrepresent PLL to support continuing limits on subsequent prescriptions, cease debate, and/or avoid blame:

It’s an easy scapegoat … And I can shut it [the conversation] down … I can move on. It takes away that I’m the bad guy. (Family Medicine MD).

Clinicians we interviewed utilized a variety of defensive medicine practices in response to PLL. In these instances, patient needs became secondary to clinicians’ own fears and concerns.

## 4. Discussion

Clinicians’ opioid-prescribing practices are affected by their beliefs that opioids are inherently risky and PLL has created too much liability, institutional responses to PLL that further restrict opioid-prescribing, mis- and re-interpretations of PLL, larger cultural views that stigmatize people who use opioids and opioid-prescribing, and a legislative response that frames opioid-prescribing as the province of law enforcement.

Thus, clinicians we interviewed align with findings that this is an era of opioid pharmacovigilance characterized by a clinical focus on the legal consequences of opioid-prescribing and fears related to patient and community harm (Crowley-Matoka & True, 2012; Hurstak et al., 2017; Knight et al., 2017). While clinicians we interviewed are concerned about patient and public safety, expressed in their desire to “do no harm,” these concerns are often mentioned cursorily. Their responses to PLL are dictated more by fears of losing their medical license, being censured by the medical board, and having to interact with law enforcement. This contrasts with Hurstak et al.’s (2017) findings that primary care practitioners’ legal fears centered on being found liable if they prescribed an opioid that directly caused patient or community harm (i.e., overdoses or deaths).

PLL may explain this difference. When PLL took effect, opioid prescription rates were decreasing for several years (Ciccarone, 2019), clinicians expressed reluctance to prescribe opioids (Hurstak et al., 2017; Knight et al., 2017), and opioids and opioid-prescribing were increasingly stigmatized (McCradden et al., 2019). Yet government and organizational stakeholders still believed legislation was necessary to stop overprescribing. This move from health policy to legislative mandate combined with uncertainty about PLL requirements, NC health administrators’ strong emphasis on legality and compliance in clinical communications about PLL (Blackburn et al., 2021), and findings that disciplinary action against outlying opioid prescribers, which occurred under the NC Safe Opioid Prescribing Initiative (Maierhofer et al., 2021) often overpowers other considerations in clinicians’ opioid-prescribing decisions (Sedney et al., 2022), likely amplified clinicians’ perception of legal risk, blunting discussions about individual patients’ needs.

This focus on avoiding liability and the ambiguous nature of pain (Crowley-Matoka & True, 2012) shifts clinicians’ work from healing to policing (Webster et al., 2019). This is supported by our findings that clinicians practice defensive medicine and stigmatize patients as potentially drug-seeking if they question opioid-prescribing practices. This sheds light on why patients who take opioids have reported opioid-prescribing legislation influences clinicians to view them with greater suspicion, stigmatize them as ‘addicts,’ and restrict patient autonomy in treatment decisions (Hurstak et al., 2017; Antoniou et al., 2019).

These practices, along with institutional policies that further restrict opioid-prescribing, clinicians’ refusal to prescribe opioids, and established routines of referring patients with pain to other providers, push patients to overuse the medical system to adequately manage their pain. Overuse contributes significantly to overall health care spending and provision (Brownlee et al., 2014; Zhang et al., 2019), harms patients physically, psychologically, financially, and practically (Satterwhite et al., 2019; Zhang et al., 2019), and leads to lower quality, fragmented care (Romano et al., 2015). These harms are heightened when patients are referred to expensive emergency departments or far-away specialists, and all patients must wait for longer periods of time as demand outstrips supply.

Some problematic elements of this process are systematic and inherent to the healthcare delivery system (e.g., lack of specialty care, clinic schedules) and existed long before PLL was enacted. However, by limiting patient autonomy and clinicians’ agency, PLL exacerbates structural problems and structural vulnerabilities (Satterwhite et al., 2019), such as underemployment (Antoniou et al., 2019).

Thus, while NC sought to address the opioid crisis by targeting clinicians’ prescribing behavior, the burden of PLL often lands on patients who clinicians report may be undertreated, viewed suspiciously, referred out for reasons unrelated to patient care, or forced to overuse the medical system.

To minimize harms, increase positive health outcomes, and improve health equity, we propose several strategies that incorporate multilevel interventions as called for by Golden and Earp (2012). First, health care laws and policies must be considered within the systems and structures in which they are implemented. PLL assumes an ideal health care system (i.e., patient care is highly accessible and patient-centered) and ideal patients (i.e., patients have few, if any barriers to access and treatments), which does not reflect the realities of U.S. health care and the multitude of forces that shape it (Stonington & Holmes, 2006). Legislative authors should realistically assess how policies will influence care and work to minimize patient barriers and structural vulnerabilities and, recognizing that health systems often do not react to improvements in predictable ways (Braithwaite, 2018), include a clear path for expeditious evaluation and amendments to allow course corrections and adjustments.

Second, given findings that PLL is reframed, defensively implemented, used by clinicians and institutions to achieve their own desired outcomes, and has resulted in the greatest decreases in opioid-prescribing rates and mean days’ supply for unintended groups, namely chronic pain patients with and without cancer (Maierhofer et al., 2023), due consideration should be given to potential misapplications and their likely impacts on clinical care and patients. Advocacy representatives warned policymakers that PLL would impact pain patients; therefore, heeding input from stakeholders and experts would be beneficial. Patient engagement is also warranted as patient engagement can lead to improvements in health services (Bombard et al., 2018).

In line with lessons learned from misapplications of the CDC’s 2016 guideline for prescribing opioids for pain, legislative clarity regarding to whom the law should *not* apply (i.e., CNCP patients) and what actions should not be taken (i.e., refusing to write second prescriptions) would be beneficial (Dowell et al., 2022). Improved communications from health organizations and professional medical societies that highlight and encourage new practices (e.g., writing a second opioid prescription), while addressing ambiguities and potential reframing of directives, could decrease unintended consequences and temper clinicians’ fears of legal liability.

Fourth, legislators, clinicians, and their organizations should support patient-centered clinical practice that is flexible, individualized, and actively engages patients in their care and treatment decisions (Braithwaite, 2018; Dowell et al., 2022). This type of care is a key element in high quality care (Epstein & Street, 2011) and helps avoid patient harms (Dowell et al., 2022). Instituting practices wherein clinicians and patients balance the benefits-risk profile of opioid therapy with each patient’s unique circumstances, including diagnosis, insurance coverage, and work/personal demands, would aid this endeavor.

Fifth, since patient engagement in clinical decision-making leads to cultural changes within organizations (Braithwaite, 2018) and clinical power dynamics being shared or neutralized (Bombard et al., 2018), patient engagement in clinical decision-making *and* organizational policy would diminish patient stigmatization and defensive medicine practices.

Patient stigmatization and its’ concomitant harms would also be lessened by clinician training in structural competency (Metzl & Hansen, 2014). Structural competency training develops clinicians’ abilities to ascertain how structural forces, including social networks, cultural zeitgeists, and political factors, give rise to clinical issues and impact clinical encounters (Metzl & Hansen, 2014). This approach combined with patient engagement would combat stigma and support patient-centered care at intrapersonal, interpersonal, institutional, and societal levels.

Finally, as social medicine maintains, it is prudent to consider the social conditions that drive opioid consumption rather than merely locating the problem in overprescribing or the drugs themselves (Dasgupta et al., 2018; Waitzkin et al., 2001; Webster et al., 2019). The rise in opioid prescriptions was fueled by structural factors (e.g., economic disadvantage, social isolation, psychological distress) (Dasgupta et al., 2018) and the lack of affordable non-opioid treatment modalities (Webster et al., 2019). Therefore, addressing these factors by, for example, expanding social support nets, paid medical leave, and insurance coverage for non-opioid pain management modalities are recommended.

This study has three limitations. Although PLL is about acute and postoperative pain, participants slipped between PLL, the larger STOP Act, opioid guidelines in general, and acute, postoperative, and chronic pain patients in their responses. They see PLL as part of a larger opioid pharmacovigilance movement (Knight et al., 2017) and struggle to tease apart the many policies. This is an important consideration for future health care legislators, policy makers, and researchers. Second, PLL came on the heels of the NC medical board’s controversial Safe Opioid Prescribing Initiative that investigated high-volume prescribers (Maierhofer et al., 2021). This may have primed clinicians to respond more defensively to PLL, honing in on liability and licensure concerns. Exploring PLL implementation and outcomes in other states would shed light on this and aid understanding regarding how clinicians implement health care legislation in various social and political milieus. Third, we did not interview patients. Patient perspectives can and do diverge from clinicians’ views (Hurstak et al., 2017). Therefore, patient interviews would enhance understanding of PLL’s downstream consequences for patients and provide insight into their conceptualizations of patient-centered care. Direct observation of clinical interactions with acute, post-operative, and CNCP patients, would illuminate how pain management decisions are made and how patients can be marginalized and centered. Examining institutional responses to health care policies and their impact on clinical practice and patient care is also warranted.
